# Gender Disparities in Secondary Patency of Arteriovenous Fistulas: Insights into Hemodialysis Outcomes and Long-Term Vascular Access Viability

**DOI:** 10.3390/jcm15051777

**Published:** 2026-02-26

**Authors:** Lutfi Çagatay Onar, Didem Melis Oztas, Murat Ugurlucan

**Affiliations:** 1 Department of Cardiovascular Surgery, Republic of Turkey, Ministry of Health, Dr. İsmail Fehmi Cumalioglu City Hospital, Tekirdag 59020, Turkey; 2 Department of Cardiovascular Surgery, Vadistanbul Liv Hospital, Istanbul 34475, Turkeymuratugurlucan@yahoo.com (M.U.)

**Keywords:** arteriovenous fistula, secondary patency, gender differences, hemodialysis, end-stage renal disease, complications, thrombosis, stenosis, vascular access, patient outcomes

## Abstract

**Background**: Autologous arteriovenous fistulas (AVFs) are the preferred vascular access modality for hemodialysis due to superior durability and lower infection rates. However, long-term secondary functional patency remains a critical determinant of dialysis success and patient survival. Although sex-related differences in vascular access outcomes have been reported, their impact on long-term secondary patency remains insufficiently clarified. **Objective**: The primary objective of this study was to evaluate long-term gender differences in the secondary functional patency of AVFs and to determine clinical, anatomical, and procedural factors contributing to these disparities. **Methods**: A retrospective cohort study including 968 ESRD patients undergoing AVF creation between 2012 and 2024 was conducted. Secondary patency was analyzed using Kaplan–Meier survival curves. Multivariate logistic regression identified independent predictors. The overall primary functional patency rates were 78.9% at 1 year, 71.2% at 3 years, and 62.7% at 5 years. When stratified by gender, male patients demonstrated primary patency rates of 80.3% at 1 year, 72.8% at 3 years, and 64.5% at 5 years, whereas female patients showed slightly lower rates of 77.3% at 1 year, 69.4% at 3 years, and 60.6% at 5 years. **Conclusions**: Female gender, particularly in the postmenopausal period, is an independent determinant of reduced long-term secondary AVF patency. Early identification of high-risk patients and structured risk factor optimization are essential to improve vascular access durability.

## 1. Introduction

Arteriovenous fistulas (AVFs) remain the gold standard for vascular access in ESRD patients due to superior long-term patency and lower complication rates compared to grafts or catheters [1,2,3]. Secondary functional patency—defined as sustained usability following salvage interventions—represents a critical clinical endpoint directly affecting dialysis continuity and patient outcomes [4,5].

Previous studies have investigated predictors of AVF maturation and primary patency [6,7,8,9]. However, long-term gender-based differences in secondary patency, particularly beyond five years, remain underexplored.

Biological differences between men and women—including vascular diameter, endothelial response, hormonal milieu, and remodeling capacity—may influence AVF durability [10]. Moreover, metabolic and cardiovascular comorbidities may interact differently across genders.

To our knowledge, this study represents one of the largest long-term analyses specifically evaluating gender disparities in secondary AVF patency while integrating anatomical, metabolic, and procedural determinants.

## 2. Materials and Methods

### 2.1. Study Design and Ethical Approval

This retrospective cohort study was conducted following approval from the institutional ethics committee (AN208.24/124) and in accordance with the principles of the Declaration of Helsinki.

The study included 968 consecutive patients diagnosed with end-stage renal disease (ESRD) who underwent primary arteriovenous fistula (AVF) creation at two similarly scaled tertiary referral centers within the same metropolitan region between January 2012 and October 2024.

Patients were eligible for inclusion if they underwent primary AVF creation and had sufficient documented follow-up to assess patency outcomes. Patients with incomplete follow-up data, early postoperative loss of access, or missing baseline anatomical measurements were excluded.

During the preparation of this manuscript, the authors used ChatGPT 5.0 (OpenAI) for language editing, structural refinement, and assistance in organizing reviewer responses. The authors critically reviewed, revised, and verified all generated content; they take full responsibility for the accuracy, integrity, and scientific validity of the final manuscript.

### 2.2. Data Collection and Clinical Variables

Demographic and clinical data were retrospectively extracted from institutional databases and patient records. Comorbid conditions, including diabetes mellitus, hypertension, hyperlipidemia, coronary artery disease, chronic obstructive pulmonary disease, and previous cerebrovascular events, were recorded. Smoking status, alcohol consumption, and glycemic control (HbA1c levels) were documented during routine nephrology follow-up.

Preoperative anatomical variables included arterial and venous diameters measured using Doppler ultrasonography. The anatomical localization of AVF creation (forearm versus upper arm) and anastomotic configuration (end-to-side or side-to-side) were systematically recorded. Secondary intervention type, frequency, and timing were also documented.

### 2.3. Definitions of Patency

Primary functional patency was defined as uninterrupted AVF use for hemodialysis without requiring surgical or endovascular intervention following initial creation. Secondary functional patency was defined as continued usability of the AVF following salvage procedures, including balloon angioplasty, thrombectomy, embolectomy, surgical revision, or reconstruction performed after initial access failure.

Patency outcomes were evaluated annually following AVF creation. Secondary patency rates were specifically assessed at six months, one year, five years, and ten years to enable standardized comparison of short- and long-term outcomes.

The interval between primary AVF creation and first re-intervention was recorded in months and analyzed as a predictor of secondary patency durability.

### 2.4. Preoperative Evaluation

Preoperative assessment included bilateral upper extremity blood pressure measurements and vascular mapping using Doppler ultrasonography.

Arterial inflow and venous outflow diameters were measured, and the access site demonstrating higher arterial pressure, larger vessel diameter, and favorable superficial venous anatomy was selected [11].

A comprehensive history of neurologic disease, prior central venous catheterization, pacemaker implantation, trauma, and previous vascular procedures was obtained [12].

### 2.5. Operative Technique

Procedures were performed predominantly under local or regional anesthesia. General anesthesia was reserved for selected cases with contraindications to regional anesthesia. During surgery, the vein was sharply dissected, and prominent branches were ligated. Approximately 2–4 cm of venous mobilization was performed to reduce tension and minimize the risk of arterial steal.

If the distal venous diameter was measured below 1.9 mm, a more proximal venous segment was selected for anastomosis [13]. Venous dilation was performed using heparinized saline or serial atraumatic vascular dilators. Arteriotomy was created using a No. 11 scalpel or Beaver blade and extended with angled scissors.

**Figure 1:**
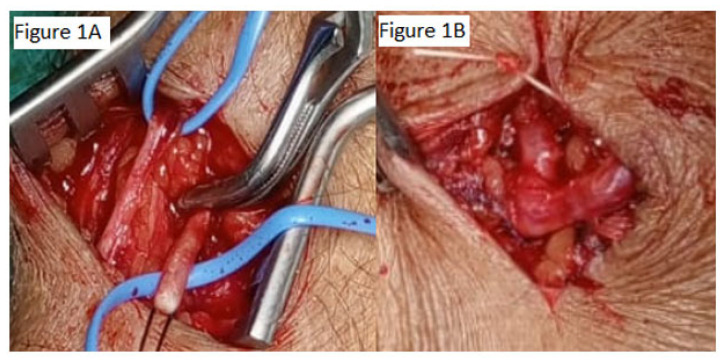
Surgical preparation and end-to-side arteriovenous fistula (AVF) creation. (**A**) Intraoperative view demonstrating vessel preparation prior to anastomosis. The radial artery (RA) and cephalic vein (CV) are identified and isolated. (**B**) Completed end-to-side (ETS) anastomosis using 7-0 polypropylene suture with fish-mouth technique. The arterial inflow segment, venous outflow tract, and anastomotic site are indicated. Abbreviations: RA, radial artery; CV, cephalic vein; ETS, end-to-side.

To reduce the risk of the arterial steal phenomenon, the arteriotomy length was limited to approximately 1.5 times the arterial diameter. End-to-side (ETS) anastomoses were constructed using 7-0 polypropylene sutures with a fish-mouth parachute technique. Side-to-side (STS) anastomosis was performed in selected cases with complex vascular anatomy (Figure 1).

Distal vein ligation was not routinely performed in order to preserve the option for guidewire access during potential future endovascular interventions.

Technical success was confirmed intraoperatively by the presence of a palpable thrill at the anastomosis site. If thrill was absent, systematic evaluation was performed to assess venous collaterals, external compression, distal flow adequacy, and anastomotic integrity. Protamine was not routinely administered in order to avoid interference with flow dynamics.

### 2.6. Postoperative Surveillance

Patients were evaluated 4–6 weeks postoperatively to assess fistula maturation. When thrill was weak or pulsatile flow predominated, Doppler ultrasonography was performed to determine the need for balloon angioplasty or surgical revision [14].

### 2.7. Secondary Interventions

In cases where AVF failed, balloon angioplasty was the preferred first-line salvage intervention. Access was obtained either via direct fistula cannulation or brachial artery approach using 0.014–0.018 inch guidewires.

Balloon diameters ranged between 4 and 12 mm with lengths up to 100 mm. Both drug-coated and uncoated balloons were used based on lesion characteristics.

Additional salvage procedures included AVF reconstruction, embolectomy, thrombectomy, and vessel-sparing surgical release of perivascular fibrotic bands.

The vessel-sparing technique involved surgical release of perivascular fibrotic bands contributing to stenosis and extrinsic compression, thereby restoring vascular compliance and optimizing hemodynamics.

Secondary patency measurement was initiated following successful completion of each salvage intervention.

### 2.8. Histopathological and Immunohistochemical Analysis

Histopathological and immunohistochemical analyses were performed in a selected subgroup of patients (n = 16) undergoing repeated reinterventions to explore structural and molecular mechanisms associated with recurrent AVF failure. Patients were selected intraoperatively based on surgical exploration findings suggestive of advanced pathological remodeling, including densely fibrotic vessel walls, severe luminal narrowing precluding intraluminal intervention, and gross structural abnormalities considered likely to compromise procedural success. Although exploratory in nature and not a predefined endpoint, these analyses provide insight into the pathological processes contributing to fistula dysfunction.

Excised AVF specimens were stained for CD3, CD31, CD46, CD55, CD20, MPO, C3d, C4d, IgA, fibrinogen, and CD71 (transferrin receptor) to evaluate immune cell infiltration, complement activation, inflammatory response, and ferroptosis-related vascular injury [15,16,17,18,19]. Due to limited specimen availability, semi-quantitative comparative analysis between failing and patent fistulas was not feasible.

### 2.9. Statistical Analysis

Statistical analyses were performed using SPSS version 26.0 (IBM Corp., Armonk, NY, USA). Continuous variables were expressed as mean ± standard deviation (SD), and categorical variables were presented as percentages. Normality of distribution was assessed using the Kolmogorov–Smirnov test. Between-group comparisons were conducted using independent-samples *t*-tests or Mann–Whitney U tests for continuous variables, and chi-square or Fisher’s exact tests for categorical variables as appropriate.

Kaplan–Meier survival analysis was employed to estimate time-dependent secondary AVF patency and overall mortality. Differences between subgroups were evaluated using the log-rank test.

To identify independent predictors of reduced secondary functional patency, multivariate logistic regression analysis was performed. An initial model including the entire cohort was constructed with gender, vessel diameter, and relevant clinical covariates entered simultaneously to evaluate their independent effects. Subsequently, gender-stratified models were developed to explore sex-specific predictors and potential differences in risk profiles between male and female patients.

Vessel diameter was included as a covariate to minimize confounding effects on patency outcomes. However, no formal interaction term between vessel diameter and biological sex was incorporated; therefore, the analysis does not directly test statistical interaction but rather assesses independent and sex-specific associations.

Covariates entered into the model included age, comorbidities, smoking status, alcohol consumption, previous history of deep venous thrombosis or stroke, the interval between primary surgery and first re-intervention, anastomotic technique, anatomical localization of the fistula, weekly dialysis frequency, and residential status.

A *p*-value <0.05 was considered statistically significant.

## 3. Results

A total of 968 patients were included in the final analysis, comprising 510 males (52.6%) and 458 females (47.4%). The mean age was 62.8 ± 8.5 years in males and 64.2 ± 7.9 years in females, with no statistically significant difference between the groups.

Hypertension was present in 678 patients (70.1%), diabetes mellitus in 432 (44.6%), hypercholesterolemia in 389 (40.2%), and coronary artery disease in 285 (29.4%). Detailed demographic, clinical, and anatomical characteristics are summarized in Table 1 and Table 2.

The mean follow-up duration for the entire cohort was 77.2 ± 15.8 months (range: 46.2–108.2 months).

**Figure 2:**
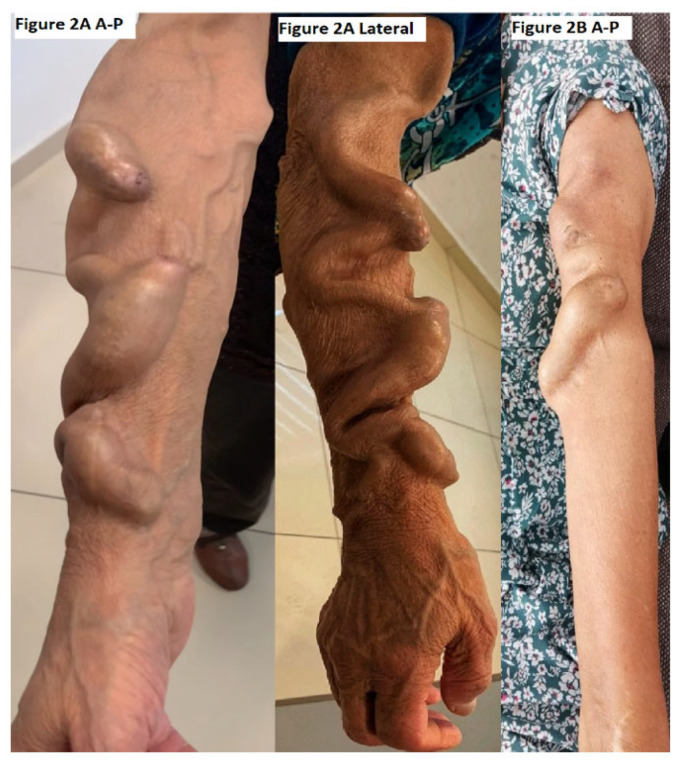
Representative cases of severe arteriovenous steal syndrome following AVF creation. (**A**) Anteroposterior (A-P) and lateral radiographic projections demonstrating markedly increased arteriovenous flow (approximately 1.3–1.0 L/min), consistent with clinically significant steal phenomenon. (**B**) A separate patient presenting with symptomatic distal ischemia associated with a high-flow AVF. Imaging findings correlate with clinical absence of distal perfusion and confirm hemodynamic compromise.

During follow-up, 11 AVFs were surgically closed due to renal transplantation (n = 4) or severe steal syndrome (n = 7) and were excluded from secondary patency analysis. Representative angiographic images of severe arteriovenous steal syndrome are presented in Figure 2A (anteroposterior and lateral views) and Figure 2B.

### 3.1. Secondary Interventions

**Figure 3:**
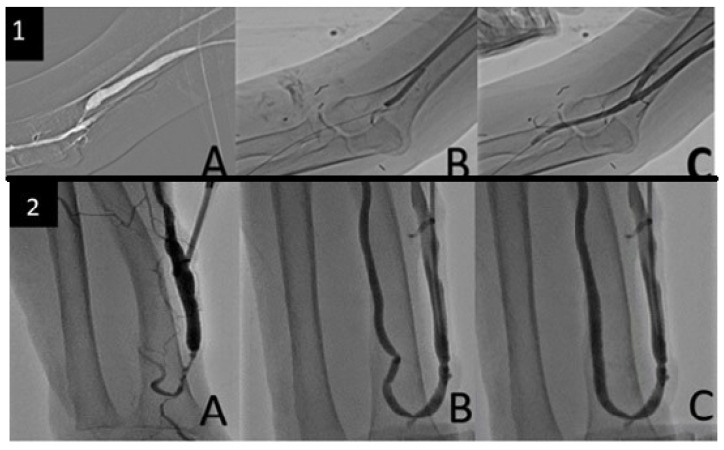
Endovascular management of recurrent AVF stenosis. (**1A**) Digital subtraction angiography (DSA) demonstrating focal juxta-anastomotic narrowing of the fistula. (**1B**) Balloon angioplasty performed across the stenotic segment. (**1C**) Post-procedural DSA showing restoration of luminal diameter and improved flow. (**2A**) Conventional angiographic image obtained via direct venous cannulation demonstrating recurrent narrowing. (**2B**) Balloon dilation revealing an additional proximal arterial stenosis. (**2C**) Final angiographic image confirming satisfactory flow restoration.

The most frequently performed re-intervention was balloon angioplasty/fistuloplasty (n = 235, 24.3%). Other secondary intervention methods are presented in Table 3. Representative angiographic images demonstrating successful balloon angioplasty and flow restoration are presented in Figure 3. No statistically significant association was observed between the type of re-intervention and long-term secondary patency outcomes.

The mean interval between primary AVF creation and first secondary intervention was 36.4 ± 12.6 months (range: 11.7–61.1 months).

### 3.2. Overall Secondary Patency

Secondary patency rates declined progressively over time. The mean secondary functional patency was 64.0% ± 5.2 at five years and 23.6% ± 4.8 at ten years.

**Figure 4:**
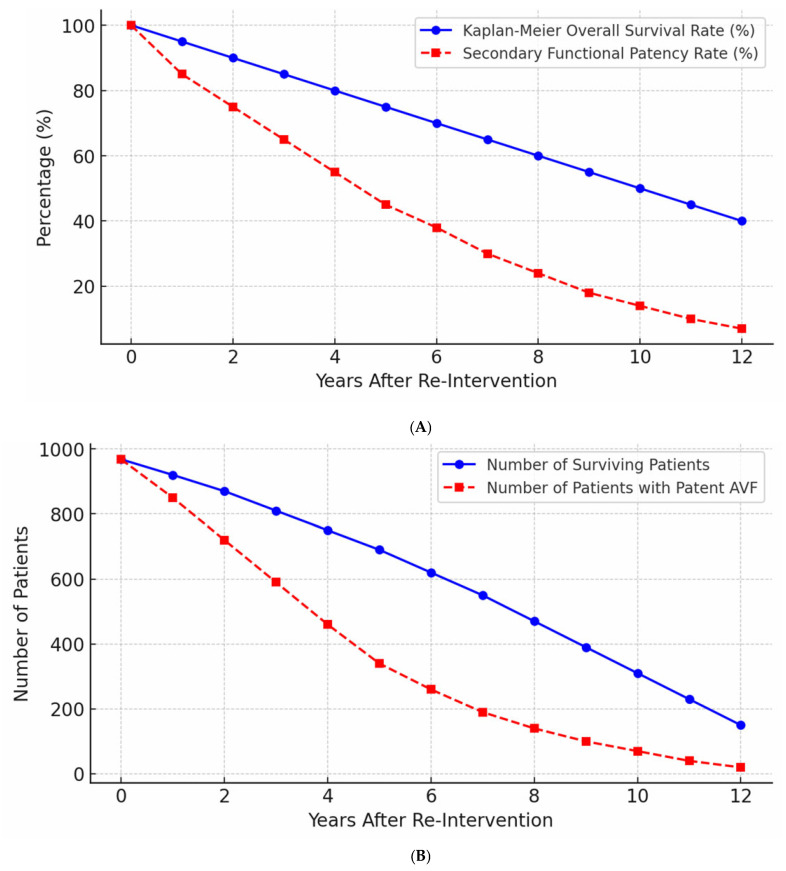
Kaplan–Meier analysis of overall survival and secondary AVF patency. (**A**) Survival probability over time in the study population. (**B**) Number of surviving patients and number of patients maintaining secondary functional patency during follow-up.

Kaplan–Meier survival curves demonstrating secondary patency are illustrated in Figure 4A. Figure 4B presents overall patient survival in parallel with secondary functional patency rates.

### 3.3. Gender-Based Secondary Patency

Gender-stratified analysis revealed significantly different long-term outcomes. Male patients demonstrated secondary patency rates of 68.4% ± 4.9 at five years and 45.6% ± 5.1 at ten years. In contrast, female patients exhibited rates of 54.1% ± 5.4 at five years and 10.0% ± 4.8 at ten years.

**Figure 5:**
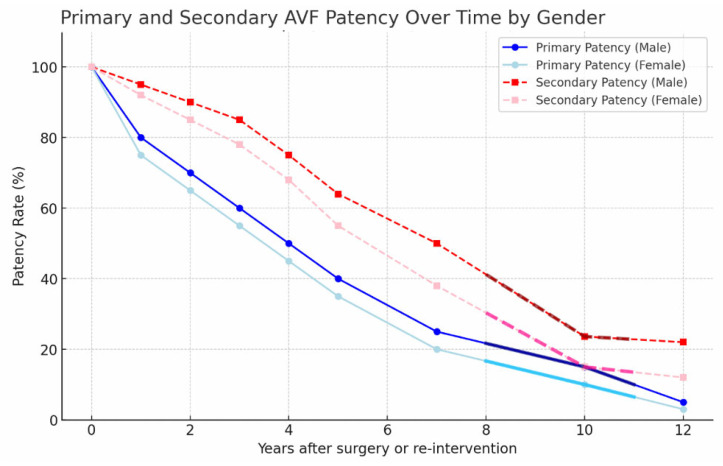
Kaplan–Meier survival curves demonstrating gender-based differences in primary and secondary functional AVF patency over long-term follow-up. Male patients exhibited significantly higher secondary patency rates compared to female patients (log-rank *p* = 0.012).

The difference between male and female patients was statistically significant at both five- and ten-year follow-up intervals (log-rank *p* = 0.012). Gender-based primary and secondary patency curves are shown in Figure 5.

### 3.4. Glycemic Control and Secondary Patency

Diabetes mellitus was associated with reduced secondary patency. Patients with HbA1c levels greater than 8.5% demonstrated significantly inferior secondary patency outcomes compared to those with lower HbA1c values (*p* = 0.018).

**Figure 6:**
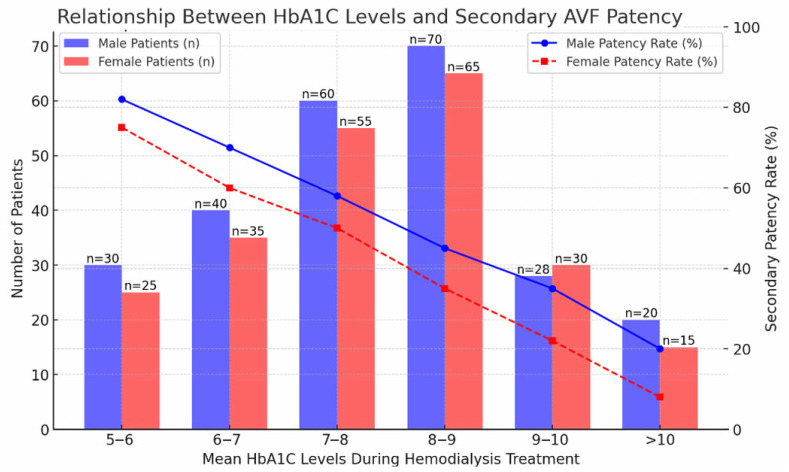
Secondary AVF patency according to glycemic control. Kaplan–Meier curves stratified by HbA1c level (≤8.5% vs. >8.5%). Poor glycemic control was associated with reduced secondary patency (*p* = 0.018).

Secondary patency according to HbA1c stratification is illustrated in Figure 6.

### 3.5. Smoking Status

Smoking status was significantly associated with secondary AVF patency (*p* = 0.005). Never-smokers demonstrated higher secondary patency rates compared to active smokers, e-cigarette users, and patients who discontinued smoking after ESRD diagnosis.

**Figure 7:**
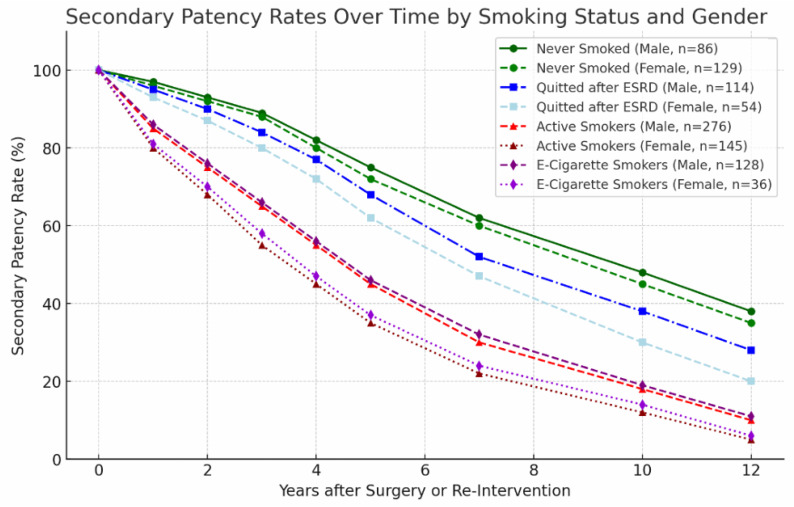
Impact of smoking status on secondary AVF patency. Kaplan–Meier curves demonstrating secondary patency among never-smokers, active smokers, e-cigarette users, and former smokers. Active smoking and e-cigarette use were associated with significantly reduced patency.

Active smokers showed significantly lower patency compared to non-smokers (*p* = 0.001), and e-cigarette users also demonstrated reduced patency (*p* = 0.02). The adverse effect of smoking was more pronounced in female patients (*p* = 0.002). The relationship between smoking patterns and secondary patency is presented in Figure 7.

### 3.6. Residence and Social Factors

Residence status significantly influenced secondary patency outcomes. Patients residing in rural areas exhibited significantly lower secondary patency compared to urban residents (*p* < 0.001).

Marital status (*p* = 0.001) and positive family history of ESRD (*p* < 0.001) were associated with improved secondary patency outcomes.

### 3.7. Premenopausal Subgroup Analysis

Improved secondary patency was observed in the premenopausal female subgroup (n = 14, 1.4% of the total cohort) compared to age-matched male patients (*p* < 0.001). This subgroup consisted of younger patients with fewer comorbidities and primarily acute renal failure etiologies.

Hormone replacement therapy was documented in 41 postmenopausal women (4.23%). No statistically significant improvement in secondary patency was observed in this subgroup when compared to premenopausal women (*p* = 0.24) or male patients (*p* = 0.40).

### 3.8. Multivariate Analysis

Multivariate logistic regression analysis identified female gender, HbA1c >8.5%, active smoking, hypertension, diabetes mellitus, coronary artery disease, frequent dialysis sessions, and shorter interval to re-intervention as independent predictors of reduced secondary patency.

The regression model stratified by gender is presented in Table 4.

### 3.9. Impact of the COVID-19 Pandemic

**Figure 8:**
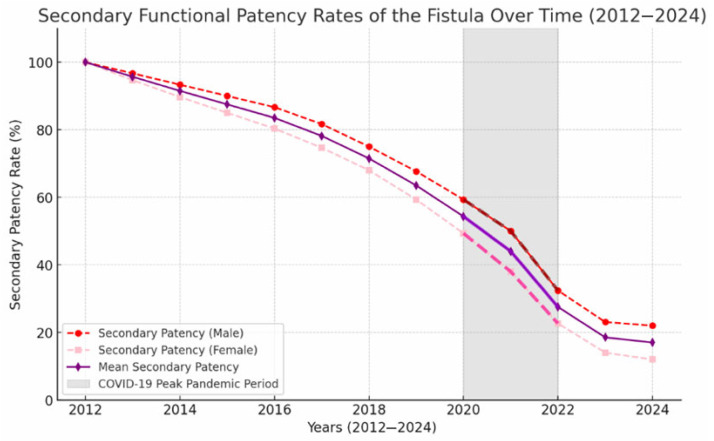
Impact of the COVID-19 pandemic on AVF patency. Kaplan–Meier curves demonstrating accelerated decline in secondary patency during the 2020–2022 period compared to pre-pandemic intervals.

A steeper decline in both primary and secondary functional patency rates was observed during the COVID-19 period (2020–2022), as demonstrated in Figure 8.

Patients with hypertension (*p* = 0.009), diabetes mellitus (*p* = 0.018), and coronary artery disease (*p* = 0.015) experienced a more pronounced decline in secondary patency during this interval.

### 3.10. Histopathological Findings

Histopathological and immunohistochemical analyses were performed in selected patients requiring multiple re-interventions.

Excised juxta-anastomotic AVF specimens demonstrated variable immunopositivity for CD3, CD31, CD20, CD71, and MPO. Increased expression of CD71 was observed in patients undergoing more than three re-interventions.

**Figure 9:**
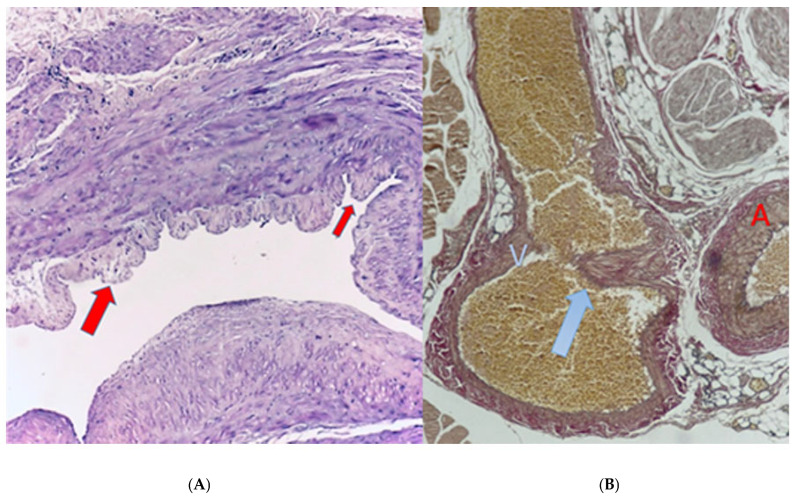
Histopathological evaluation of recurrent AVF failure. (**A**) Histological section of an excised AVF from a high-risk female patient demonstrating marked venous intimal hyperplasia. (**B**) Oblique cross-sectional microscopy of an end-to-side anastomosed fistula revealing severe arterial intimal hyperplasia extending to the anastomotic site, resulting in near-total luminal narrowing.

Representative histopathological findings are illustrated in Figure 9A,B.

## 4. Discussion

Our study was designed to evaluate gender-dependent determinants of long-term secondary AVF patency and to identify potentially modifiable clinical variables influencing vascular access durability. The principal finding of this study is that female gender independently predicts reduced long-term secondary patency, particularly in the postmenopausal period. This finding was consistent across five- and ten-year follow-up analyses (Figure 5) and remained significant in multivariate modeling (Table 4). Given the extremely limited sample size of this subgroup, this observation should be interpreted as hypothesis-generating rather than confirmatory evidence of estrogen-mediated protection.

Although autologous AVFs remain the preferred vascular access modality [20,21,22], gender-based disparities persist. Our data demonstrated significantly lower secondary patency in female patients compared to males (log-rank *p* = 0.012), supporting prior observations that women with ESRD may exhibit impaired vascular remodeling and increased susceptibility to thrombosis [21,22]. Unlike previous studies primarily focused on primary patency or early maturation, our analysis extends these observations to long-term secondary patency beyond five years.

Glycemic control emerged as a clinically relevant determinant. As demonstrated in Figure 6, patients with HbA1c levels greater than 8.5% exhibited significantly inferior secondary patency outcomes (*p* = 0.018). These findings are consistent with the established association between poor glycemic control, endothelial dysfunction, and accelerated neointimal hyperplasia [23]. The interaction between diabetes and female gender may further amplify adverse remodeling processes.

Dialysis frequency was also associated with reduced patency. Patients requiring more frequent sessions demonstrated poorer long-term outcomes, likely reflecting advanced systemic disease burden. Previous studies have reported that repetitive endothelial stress and altered hemodynamics contribute to thrombosis and intimal proliferation [24,25], which may explain the observed decline.

Percutaneous balloon angioplasty was the most frequently utilized salvage procedure (Table 3). Although intervention type was not independently associated with long-term secondary patency, angioplasty demonstrated high immediate technical success rates. These findings are aligned with current literature supporting endovascular approaches as first-line management for stenotic lesions [26,27,28,29,30,31].

Smoking status was a strong modifiable predictor of reduced patency (Figure 7). Active smokers and e-cigarette users exhibited significantly inferior outcomes (*p* = 0.005), with a more pronounced effect observed in female patients (*p* = 0.002). These findings corroborate prior evidence linking smoking to endothelial injury, nitric oxide dysregulation, vascular calcification, and thrombosis [23]. The data reinforce the importance of aggressive smoking cessation strategies in patients undergoing AVF creation.

Anatomical variables likely contribute to gender disparities. As shown in Table 2, male patients exhibited larger arterial and venous diameters. Vessel diameter influences postoperative remodeling capacity and flow adaptation [32,33,34,35,36]. While venous diameter alone may not fully predict long-term maturation, smaller arterial inflow may limit adaptive remodeling and increase thrombosis susceptibility [37]. These anatomical differences may partially explain the reduced secondary patency observed in female patients. Although vessel diameter was included in the regression model, the independent association of female gender persisted, suggesting that anatomical size alone does not fully explain the observed disparity.

The COVID-19 pandemic period (2020–2022) was associated with a steeper decline in secondary patency rates (Figure 8). Similar findings have been reported globally, highlighting the impact of delayed interventions and healthcare disruptions in dialysis populations [38]. The decline observed during the pandemic period is more likely related to healthcare access disruption and delayed interventions rather than direct biological thrombotic mechanisms, as serological data were not available. Our data emphasize the importance of uninterrupted vascular access surveillance even during public health crises.

Residence status also influenced outcomes. Rural patients exhibited significantly lower secondary patency (*p* < 0.001), consistent with prior reports demonstrating disparities in access to specialized vascular care [39,40]. Geographic barriers and delayed referral may contribute to these findings.

Histopathological evaluation of patients requiring multiple re-interventions revealed increased CD71 expression in selected cases, suggesting enhanced ferroptosis-related activity [17]. Increased expression of the transferrin receptor CD71 observed in selected specimens may suggest possible involvement of ferroptosis-related pathways in recurrent arteriovenous fistula failure; however, these findings remain exploratory and require further mechanistic investigation.

Overall, this study provides a comprehensive long-term evaluation of gender-specific differences in secondary AVF patency. By integrating clinical, anatomical, metabolic, and procedural variables, our findings support individualized vascular access planning and early risk factor optimization, particularly in high-risk female patients. Emerging device-based strategies aimed at improving AVF maturation, including perivascular drug delivery systems and bioengineered vascular scaffolds, represent promising translational advances. Identification of gender-specific remodeling patterns may inform device development and patient selection strategies [39,40,41,42,43,44].

## 5. Limitations

This study has several limitations that should be acknowledged. First, its retrospective design may introduce selection bias and limit causal inference. Although consecutive patients were included, unmeasured confounding variables may have influenced long-term patency outcomes.

Second, the study was conducted in two centers within the same metropolitan region, which may limit generalizability to broader populations with different healthcare infrastructures. Variations in nephrology practice patterns and access to vascular expertise across regions were not fully captured.

Third, pharmacologic data such as antiplatelet therapy adherence, anticoagulant use, and detailed lipid management strategies were not uniformly available throughout the entire follow-up period. These factors may influence vascular remodeling and thrombosis risk.

The premenopausal female subgroup represented a small proportion of the total cohort, which limits the strength of conclusions regarding hormonal influence. Similarly, histopathological and immunohistochemical analyses were performed in selected patients and were exploratory in nature.

Because a unified regression model including interaction testing between gender and vessel diameter was not performed, the independent biological contribution of sex beyond anatomical factors cannot be definitively established.

Despite these limitations, the study benefits from a large sample size, extended follow-up duration, and comprehensive evaluation of clinical, anatomical, and procedural variables affecting secondary AVF patency.

## 6. Conclusions

This study demonstrates that female gender is independently associated with significantly reduced long-term secondary arteriovenous fistula patency, particularly in the postmenopausal period. Poor glycemic control, active smoking, advanced cardiovascular dysfunction, and increased dialysis frequency further contribute to patency deterioration.

These findings emphasize the importance of individualized vascular access planning, structured surveillance, and aggressive optimization of modifiable risk factors to improve long-term hemodialysis access durability. Early identification of high-risk patients may enhance clinical outcomes and reduce repeated intervention burden.

## Figures and Tables

**Table 1:** Baseline demographic and clinical characteristics of the study population.

Parameter	Male n = 512 (52.6%)	Female n = 456 (47.4%)
**Age (Mean ± SD)**	63.8 (±14.1)	63.2 (±19.3)
**Hypertension**	**359** (70.1%)	**319** (70.0%)
**Diabetes Mellitus**	**228** (44.5%)	**204** (44.7%)
**Hypercholesterolemia**	**195** (38.1%)	**194** (42.5%)
**Coronary artery disease**	**246** (48.0%)	**291 (63.8%)**
**Smoking status (n,%)**		
***Never Smoked***	**112 (21.9%)**	103 (22.6%)
***Quitted Smoking***	86 (16.8%)	82 (18.0%)
***Active Smokers***	210 (41.0%)	211 (46.3%)
***E-Cigarette Smokers***	104 (20.3%)	60 (13.2%)
**Marital Status (n, %)**		
***Married***	**374 (73.0%)**	341 (74.8%)
***Single***	**104** (20.3%)	**96 (21.05%)**
***Widow***	**34 (6.6%)**	**19 (4.1%)**
**Residence Status (n, %)**		
***Rural***	147 (28.7%)	132 (28.9%)
***Urban***	365 (71.3%)	324 (71.1%)
**Family History of CRD (n, %)**	**102** (19.9%)	96 (21.1%)

Data included age, gender distribution, comorbid conditions, smoking status, and relevant laboratory parameters.

**Table 2:** Preoperative anatomical characteristics and anastomotic configurations.

Male		Female		
**Mean Venous Diameter**	**(mm, Mean ± SD)**	***p*** **-Value**	**(mm, Mean ± SD)**	***p*** **-Value**
**Forearm**	**2.6 (±0.5)**	<0.001	**1.9 (±0.4)**	0.550
**Upper arm**	**4.0 (±0.6)**	<0.001	**3.1 (±0.5)**	<0.001
**Mean Arterial Diameter**	**(mm, Mean ± SD)**		**(mm, Mean ± SD)**	
**Forearm**	**3.0 (±0.6)**	0.250	**2.3 (±0.5)**	0.150
**Upper arm**	**4.2 (±0.7)**	<0.001	**3.5 (±0.5)**	<0.001
**Technique**				
** End-to-Side**	**426 (83.2 %)**	<0.001	**315 (69.1 %)**	0.620
** Side-to-Side**	**86 (16.8 %)**	0.620	**141 (30.9 %)**	<0.001

Data included arterial and venous diameters, anatomical location of AVF creation (forearm vs. upper arm), and anastomotic technique (ETS vs. STS).

**Table 3:** Distribution and frequency of secondary intervention types.

Procedure	Number (n)	Proportion (%)	Mean Success Rate
Balloon Angioplasty/Fistuloplasty	**235**	**24.31**	**86.5% (±5.16)**
AVF creation at the same anatomical region	**200**	**20.64**	**84.64% (±7.70)**
AVF creation in other anatomical region	**178**	**18.41**	**86.01% (±2.10)**
Reconstruction of the previous AVF	**142**	**14.65**	**74.10% (±7.02)**
Embolectomy/Thrombectomy	**115**	**11.88**	**72.12% (±4.46)**
Vessel sparing angioplasty	**98**	**10.12**	**64.16% (±5.85)**

Includes balloon angioplasty, thrombectomy, embolectomy, AVF reconstruction, and re-creation in alternative anatomical sites.

**Table 4:** Multivariate logistic regression analysis of independent predictors of reduced secondary AV.

Variable	Female Gender			Male Gender		
	Odds Ratio (OR)	95% Confidence Interval (CI)	*p*-Value	Odds Ratio (OR)	95 % Confidence Interval (CI)	*p*-Value
**Age (<50 years)**	1.05	0.78–1.32	0.550	1.02	0.80–1.29	0.710
**Age (>65 years)**	1.92	1.45–2.48	<0.001	1.12	0.90–1.42	0.250
**Diabetes**	1.76	1.38–2.24	<0.001	1.48	1.15–1.90	0.002
**HbA1C (>8.5%)**	2.85	2.30–3.55	<0.001	2.10	1.65–2.72	<0.001
**Active Smoking**	1.65	1.25–2.12	<0.001	1.45	1.12–1.87	0.005
**Hypertension**	1.18	0.90–1.52	0.150	1.12	0.86–1.45	0.390
Mild *LVSD (LVEF <40%)	2.42	1.85–3.10	<0.001	1.15	0.92–1.43	0.210
Severe *LVSD (LVEF <30%)	3.12	2.40–3.89	<0.001	2.05	1.55–2.68	<0.001
**NYHA 1**	1.08	0.89–1.35	0.320	1.05	0.85–1.30	0.640
**NYHA 2**	1.58	1.22–2.05	<0.001	1.09	0.86–1.38	0.430
**NYHA 3**	2.04	1.65–2.54	<0.001	1.20	0.95–1.52	0.150
**NYHA 4**	3.21	2.52–3.98	<0.001	2.42	1.90–3.05	<0.001
**COPD	1.38	1.05–1.82	0.030	1.30	1.02–1.67	0.035
**BMI >30**	1.14	0.89–1.50	0.260	1.08	0.86–1.35	0.480
**Alcohol Consumption**	1.80	1.35–2.32	<0.001	1.62	1.22–2.12	<0.001
**Previous History of DVT**	1.30	1.00–1.72	0.055	1.18	0.89–1.55	0.290
**Previous History of Stroke**	2.11	1.60–2.78	<0.001	1.25	0.98–1.61	0.070
**Operation Time (>1 Hour)**	1.10	0.88–1.39	0.290	1.30	1.02–1.65	0.045
**Time Interval Between the first surgery to re-intervention (<1 Month)**	2.25	1.70–2.97	<0.001	1.75	1.35–2.29	<0.001
**Time Interval Between the first surgery to re-intervention (>12 Months)**	1.08	0.85–1.40	0.390	1.15	0.89–1.50	0.250
**Dialysis Sessions (>2 per week)**	2.30	1.80–2.95	<0.001	2.05	1.62–2.70	<0.001
**Dialysis Sessions (<2 per week)**	1.10	0.85 –1.42	0.420	1.05	0.80–1.40	0.620

F patency stratified by gender. Includes adjusted odds ratios (OR), 95% confidence intervals (CI), and *p*-values. * Left Ventricular Systolic Dysfunction. ** Chronic Obstructive Lung Disease.

## Data Availability

No new data were created or analyzed in this study.
